# Favorable clinical outcome following surgical evacuation of deep-seated and lobar supratentorial intracerebral hemorrhage: a retrospective single-center analysis of 123 cases

**DOI:** 10.1007/s00701-018-3622-9

**Published:** 2018-07-26

**Authors:** Amel Hessington, Parmenion P. Tsitsopoulos, Andreas Fahlström, Niklas Marklund

**Affiliations:** 1 Department of Neuroscience, Section of Neurosurgery, Uppsala University Hospital, Uppsala, Sweden; 2 Hippokratio General Hospital, Aristotle University, Thessaloniki, Greece; 3 Department of Clinical Sciences Lund, Neurosurgery, Skåne University Hospital, Lund University, Lund, Sweden

**Keywords:** Intracerebral hemorrhage, Mortality, Outcome, Prognostic factors, Craniotomy, Surgery

## Abstract

**Background:**

In spontaneous supratentorial intracerebral hemorrhage (ICH), the role of surgical treatment remains controversial, particularly in deep-seated ICHs. We hypothesized that early mortality and long-term functional outcome differ between patients with surgically treated lobar and deep-seated ICH.

**Method:**

Patients who underwent craniotomy for ICH evacuation from 2009 to 2015 were retrospectively evaluated and categorized into two subgroups: lobar and deep-seated ICH. The modified Rankin Scale (mRS) was used to evaluate long-term functional outcome.

**Result:**

Of the 123 patients operated for ICH, 49.6% (*n* = 61) had lobar and 50.4% (*n* = 62) deep-seated ICH. At long-term follow-up (mean 4.2 years), 25 patients (20.3%) were dead, while 51.0% of survivors had a favorable outcome (mRS score ≤ 3). Overall mortality was 13.0% at 30 days and 17.9% at 6 months post-ictus, not influenced by ICH location. Mortality was higher in patients ≥ 65 years old (*p* = 0.020). The deep-seated group had higher incidence and extent of intraventricular extension, younger age (52.6 ± 9.0 years vs. 58.5 ± 9.8 years; *p* < 0.05), more frequently pupillary abnormalities, and longer neurocritical care stay (*p* < 0.05). The proportion of patients with good outcome was 48.0% in deep-seated vs. 54.1% in lobar ICH (*p* = 0.552). In lobar ICH, independent predictors of long-term outcome were age, hemorrhage volume, preoperative level of consciousness, and pupillary reaction. In deep-seated ICHs, only high age correlated significantly with poor outcome.

**Conclusions:**

At long-term follow-up, most ICH survivors had a favorable clinical outcome. Neither mortality nor long-term functional outcome differed between patients operated for lobar or deep-seated ICH. A combination of surgery and neurocritical care can result in favorable clinical outcome, regardless of ICH location.

## Introduction

Spontaneous intracerebral hemorrhages (ICH) constitute 9–27% of all strokes worldwide [11], and are significant causes of morbidity and mortality with a 30-day mortality of about 40–54% at 1 year [13, 31, 45]. Despite ongoing attempts to develop effective medical therapies and optimize surgical interventions, indications for surgical treatment have not been clearly defined and remain controversial [17, 41, 42]. Regardless of best medical management and/or surgery, functional outcome remains poor [34, 37] since less than 20% of all ICH patients have regained functional independence at 6 months [26]. Spontaneous supratentorial ICH account for the majority of all ICHs [10, 15] where deep-seated ICH in locations such as basal ganglia or thalamus differ clinically from superficial, lobar bleedings [12]. Nevertheless, the influence of ICH location on outcome remains unclear [16, 32, 35].

For decades, the role of surgery in ICH and whether hemorrhage evacuation can improve clinical outcome has been a topic of intense debate. The rationale for ICH surgery is that clot removal might reduce tissue damage, possibly by relieving local ischemia and by removing factors noxious to the surrounding tissue, as well as by reducing elevations of intracranial pressure (ICP) [2, 18]. Moreover, surgical access to the hemorrhage may also inflict tissue injury caused by the trajectory through uninjured brain tissue, especially in deep-seated ICHs, and from the ICH removal per se. Nevertheless, in selected patients with large hemorrhages and rapid progression of neurological deficits, surgical evacuation can be lifesaving [17, 41].

Existing randomized controlled trials (RCTs) have not clearly established a clinical benefit for surgical evacuation in ICH patients. In the STICH trial, a multicenter trial that used clinical equipoise as inclusion criterion, a subgroup analysis suggested a surgical benefit only in superficial lobar, although not in deep-seated ICHs [27]. In fact, a worse outcome following surgery compared to best medical management was seen in comatose patients with deep-seated ICHs. In the follow-up STICH II trial, a small but significant improvement was observed following surgery for lobar ICHs evacuated within 21 h after onset of symptoms [28]. In another trial that included 108 patients with subcortical and putaminal ICHs, surgically treated patients had a better functional outcome than conservatively treated controls [30]. In conclusion, the lack of sufficiently robust evidence has made it difficult to establish clear criteria for ICH evacuation based on the location [17, 41, 42].

We hypothesized that ICH location (lobar vs. deep-seated) would influence clinical outcome, mortality, and long-term functional outcome, in patients treated by craniotomy and ICH evacuation.

## Material and methods

### Patients and setting

The study was approved by the Regional Research Ethical Review Board of Uppsala University. All patients > 18 years old who underwent surgical evacuation for spontaneous supratentorial ICH at the Department of Neurosurgery, Uppsala University Hospital, Uppsala, Sweden, between 2009 and 2015 were retrospectively reviewed. The department of Neurosurgery at Uppsala University Hospital is the only hospital providing neurosurgical treatment in a defined health care region covering a catchment area of over 2 million inhabitants. In Sweden, the annual ICH incidence is estimated to be 28/100000 inhabitants with higher rates in males and in the elderly population [29]. The total number of estimated ICH patients in our region based on this number and using data from the Swedish National Board of Health and Welfare are 573 per year and 4010 during the entire period used in the present study [39]. In 2017, 7% of all ICH patients included in the Riksstroke—The Swedish Stroke Register—in our region were surgically treated [33], a number that also encompasses ICH treated with only an external ventricular drainage, or cerebellar hemorrhages.

Sixteen patients with ICH due to tumor, trauma, or vascular malformation were excluded. Those with subarachnoid, subdural, cerebellar, or brain stem hemorrhages as well as pediatric patients were also not included. All surgical and clinical decisions were made by a consultant neurosurgeon on an individual basis. In general, candidates for surgical ICH evacuation were patients presenting with signs of impaired or deteriorating level of consciousness and a surgically accessible ICH. Elderly patients, typically > 75–80 years old, with or without significant co-morbidities were not considered surgical candidates. In addition, patients deeply comatose, GCS 3–5, on presentation or bilaterally unresponsive pupils were also not considered candidates for surgery. Time to surgery was defined as time from known or presumed ictus to surgery.

The ICH was evacuated by craniotomy using a free bone flap, followed by clot evacuation using a microneurosurgical technique. Postoperatively, patients were managed in the Neurocritical Care (NCC) unit with a standardized treatment protocol focusing on the monitoring, detection, and treatment of secondary insults such as increased ICP and reduced cerebral perfusion pressure (CPP) [9, 43].

### Radiological evaluation

A non-contrast head computerized tomography (CT) scan was done in all patients on admission. The CT images were evaluated by a researcher (AH) who had no previous knowledge of the patients’ clinical history. For each patient, every CT scan was analyzed for hemorrhage size, side, and location, as well as midline shift and extent of intraventricular hemorrhage (IVH). The ICH location was considered deep-seated when the hemorrhage originated or involved predominantly the basal ganglia or the thalamus. An ICH was classified as lobar, when the hemorrhage was predominantly located in the cortex and underlying white matter of the cerebral hemisphere [35].

ICH volume was measured using the validated A × B × C/2 method: height × length × width/2 [24]. Intraventricular hemorrhage volume was not included in the ICH volume measurement. The degree of intraventricular hemorrhage was analyzed using the Graeb score, a semiquantitative score ranging from 0 to 12. According to this scale, “0” refers to no visible blood in any ventricle. A maximum score of “4” is given for each lateral ventricle where it is expanded and filled with blood and “2” when the 3rd and 4th ventricles are filled in a similar way [19].

### Outcome measures

For the estimation of neurological and functional status at discharge, the motor component of the Glasgow Coma Scale (mGCS) was used. A standardized postal questionnaire was sent to every patient alive and used for the assessment of clinical outcome according to the modified Rankin Scale (mRS) [46]. If no reply was received, patients or their relatives were contacted by phone and the questionnaires could be completed by a structured telephone-based interview [43, 44]. Length of survival in those deceased at follow-up was obtained from medical records and archives. Specific cause of death was not registered. An mRS score of 0–3 was considered a favorable outcome, while poor outcome was defined as an mRS score 4–6. Those categorized in the poor outcome group were either severely disabled and unable to walk without assistance (dependent), bedridden with incontinence requiring nursing care, or dead [4].

### Statistical analysis

Continuous variables were presented either as mean (SD) or median (IQR 25th–75th percentile) values. Categorical variables were presented as numbers and percentages and compared using chi-square test. Normally distributed data were compared between groups using Student’s *t* test. The Mann Whitney’s *U* test was used to compare non-normally distributed data. Statistical significance was set at *p* < 0.05 upon 2-sided testing. Kaplan-Meier curve was used to estimate cumulative mortality.

Variables related to long-term functional outcome with *p* < 0.05 in the univariate analysis were further analyzed using binary logistic regression*.* Dichotomized (mRS ≤ 3 and mRS > 3) outcome was used as dependent variable. To determine the final independent predictive role, variables associated with poor functional outcome were incorporated into a multivariate model using standard regression analysis. Results are reported as odds ratio (OR) with a 95% confidence interval (CI).

A propensity score matched analysis was used to reduce the selection bias and potential baseline differences between the lobar and deep-seated ICH group. Propensity scores were computed using a logistic regression model, in which the dependent variable was whether the patient had lobar or deep-seated bleeding and the independent variable tested was age. The method used was 1:1 nearest-neighbor matching, with caliper width 0.2 to avoid pairing dissimilar individuals [3]. As a result, two age-matched groups with 44 patients in each group were obtained. The age-matched groups were then compared regarding early mortality and long-term outcome.

IBM SPSS Statistics version 23 (IBM, Armonk, NY, USA) was used for data analysis.

## Results

### Baseline characteristics

Demographic characteristics are presented in Table 1. A total of 123 patients with surgically treated ICH were identified. Based on the ICH incidence during the study period in the catchment area of our hospital, 3.1% of all ICH patients  were subjected to neurosurgical treatment by open craniotomy and ICH evacuation. The average age was 55.5 ± 9.8 years (range 21–76 years) with more patients being ≥ 65 years old in the lobar ICH group compared to the deep ICH (lobar, 18/61 [29.5%]; deep, 3/62 [4.8%]; *p* < 0.001). Arterial hypertension (> 140/90 mmHg) was the most common risk factor, with 41 (33.3%) patients on antihypertensive therapy. Median mGCS score was 5.0 (IQR 5–6) immediately prior to surgery and 83 patients (67.5%) were unconscious (mGCS ≤ 5 or lower) (Table 2). Eight patients (6.5%) underwent re-evacuation of the hemorrhage (Table 2), of whom five (62.5%) were on anticoagulants or antiplatelet drugs prior to ICH onset.

**Table 1:** Baseline characteristics of patients on admission

Characteristics	All (*n* = 123)	Deep ICH (*n* = 62)	Lobar ICH (*n* = 61)	*p* value
Age	55.5 ± 9.8	52.6 ± 9.0	58.5 ± 9.8	*< 0.001*
< 65	102 (82.9%)	59 (95.2%)	43 (70.5%)	*< 0.001*
Gender				
Female	41 (33.3%)	20 (32.3%)	21 (34.4%)	0.850
Vascular risk factors				
Hypertension	64 (52.0%)	31 (50.0%)	33 (54.1%)	0.649
Diabetes mellitus	9 (7.3%)	6 (9.7%)	3 (4.9%)	0.311
Excessive alcohol consumption	18 (14.6%)	11 (17.7%)	7 (11.5%)	0.326
Pupillary abnormalities* (*n*)	72 (58.5%)	42 (67.7%)	30 (49.2%)	*0.015*
MABP on admission (mmHg)	120.0 ± 23.5	124.6 ± 25.6	115.3 ± 20.4	*0.027*
Blood glucose on admission (mmol/l)	8.3 ± 2.8	8.6 ± 3.1	8.2 ± 2.5	0.483
BMI (kg/m2)	27.5 ± 7.2	28.3 ± 8.6	26.8 ± 6.2	0.308
Medical history				
Prior stroke	20 (16.3%)	5 (8.1%)	15 (24.6%)	*0.013*
Prior myocardial infarction	10 (8.1%)	6 (9.7%)	4 (6.6%)	0.527
Atrial fibrillation	8 (6.5%)	3 (4.8%)	5 (8.2%)	0.450
Other medical disorders	43 (35.0%)	17 (27.4%)	26 (42.6%)	0.077
Medication at time of ICH				
Anticoagulants	13 (10.5%)	4 (6.5%)	9 (14.8%)	0.134
Antiplatelet medication	27 (22.0%)	13 (21.0%)	14 (23.0%)	0.790
Antihypertensive medication	41 (33.3%)	16 (25.8%)	25 (41.0%)	0.074

For continuous variables, data are presented as mean ± SD; for categorical variables, data are presented as numbers and percentages (%). A *p*-value <0.05 is indicated by italics

*BMI* body mass index, *ICH* intracerebral hemorrhage, *MABP* mean arterial blood pressure

*Denotes unequal pupil size, pathological pupillary light response, miosis, or mydriasis

**Table 2:** Preoperative clinical picture and post-operative neurocritical care features

Characteristics	All (*n* = 123)	Deep ICH (*n* = 62)	Lobar ICH (*n* = 61)	*p* value
mGCS immediately preoperatively	5 (5–6)	5 (5–5)	5 (5–6)	0.066
1	1 (1%)	0 (0%)	1 (2%)	0.311
2	3 (3%)	1 (2%)	2 (3%)	0.549
3	6 (5%)	2 (3%)	4 (7%)	0.391
4	14 (11.4%)	12 (19.4%)	2 (3.3%)	*0.005*
5	59 (48.0%)	32 (51.6%)	27 (44.3%)	0.415
6	40 (32.5%)	15 (24.2%)	25 (41.0%)	*0.047*
Time from ictus to surgery (h)	13 (8–30)	12 (7–22)	18 (8–45)	*0.050*
External ventricular drainage (*n*)	62 (50.4%)	42 (67.7%)	20 (32.8%)	*< 0.001*
Period with EVD (days)	5.0 ± 6.1	6.7 ± 6.2	3.3 ± 5.5	*0.002*
Days of EVD drainage (*n*)	1.8 ± 3.3	2.7 ± 3.7	0.9 ± 2.6	*0.003*
Mechanical ventilation (days)	7.2 ± 6.7	8.5 ± 6.5	5.9 ± 6.6	*0.031*
Tracheotomy	35 (28.5%)	22 (35.5%)	13 (21.3%)	0.082
Re-evacuation	8 (6.5%)	4 (6.5%)	4 (6.6%)	0.981
Length of stay (days)	14.0 ± 10.0	15.6 ± 11.2	12.3 ± 8.3	0.068

For continuous variables, data are presented as mean ± SD and median (IQR); for categorical variables, data are numbers and percentages (%). A *p*-value < 0.05 is indicated by italics

*mGCS* motor component of Glasgow Coma Scale score, *EVD* external ventricular drainage

Although patients with deep and lobar ICH shared numerous similar baseline characteristics, those with deep ICH were younger (58.5 ± 9.8 years in lobar ICH vs. 52.6 ± 9.0 in deep ICH, *p* < 0.001), had higher mean arterial blood pressure (MABP) on arrival at the hospital (125 ± 26 vs. 115 ± 21; *p* = 0.027), and had more frequently pupillary abnormalities (*p* = 0.015). On the other hand, individuals with lobar ICH had a more frequent history of prior stroke (*p* = 0.013), but were more likely to be conscious prior to surgery (*p* = 0.047).

### Neuroradiology

From the CT scans, 61 cases (49.6%) were defined as lobar and 62 (50.4%) as deep-seated hemorrhages. Of all deep ICHs, one patient had thalamic hemorrhage while the remaining were located in the basal ganglia. There was an even distribution of left- vs. right-sided ICHs (Table 3). The mean preoperative hemorrhage volume of all patients was 84.8 mL and 80 patients (65.0%) also had IVH. Slightly larger mean hemorrhage volume was noted in patients with lobar compared to those with deep-seated hemorrhages (88.3 ± 32.9 ml vs. 81.3 ± 39.7 ml; *p* = 0.295). However, patients with deep-seated hemorrhages showed a higher degree of IVH (*p* = 0.002) and more frequently intraventricular extension (*p* = 0.032).

**Table 3:** Radiological characteristics of intracerebral hemorrhage

Characteristics	All (*n* = 123)	Deep ICH (*n* = 62)	Lobar ICH (*n* = 61)	*p* value
Side of hemorrhage	Left 65 (52.8%)	Left 34 (54.8%)	Left 31 (50.8%)	0.655
Hemorrhage volume (ml)	84.8 ± 35.6	81.3 ± 39.7	88.3 ± 32.9	0.295
Depth from cortical surface (mm)	6.2 ± 6.7	11.5 ± 5.7	1.0 ± 1.6	*0.001*
Intraventricular extension (*n*)	80 (65.0%)	46 (74.2%)	34 (55.7%)	*0.032*
Degree of IVH٭	2 (0–5)	3 (0–6)	1 (0–2)	*0.002*

For continuous variables, data are presented as mean ± SD; for categorical variables, data are presented as number and percentages (%). Values in italics represent statistically significant values (*p* < 0.05)

*IVH* intraventricular hemorrhage, *ICH* intracerebral hemorrhage

٭Graeb score

### Treatment and surgical data

All patients needed neurocritical care (NCC). Median time from onset of symptoms to surgery was 13.0 h (IQR 8–30; range 2–331 h), and 86 patients (69.9%) were operated within 24 h from ictus. An external ventricular drainage (EVD) was used for cerebrospinal fluid (CSF) drainage and ICP monitoring in 62 patients (50.4%). The EVD remained in place for 5.0 ± 6.1 days and was used for CSF drainage for 1.8 ± 3.3 days. During the immediate postoperative period, three patients developed cerebral infarcts, five a culture-verified meningitis, 40 a ventilator-associated pneumonia, and one patient a pulmonary embolus.

Patients with deep-seated hemorrhages were operated earlier (deep, 12 h [IQR 7–22]; lobar, 18 h [IQR 8–45]; *p* = 0.050), but had ventilatory support for a longer period compared to patients with lobar ICH (*p* = 0.031). In the deep ICH group, more patients received ventriculostomy (42 [67.7%] vs. 20 [32.8%]; *p* < 0.001) with longer duration of CSF drainage compared to lobar ICH patients (2.7 ± 3.7 vs. 0.9 ± 2.6 days; *p* = 0.003; Table 2).

### Mortality and long-term functional outcome

**Fig. 1:**
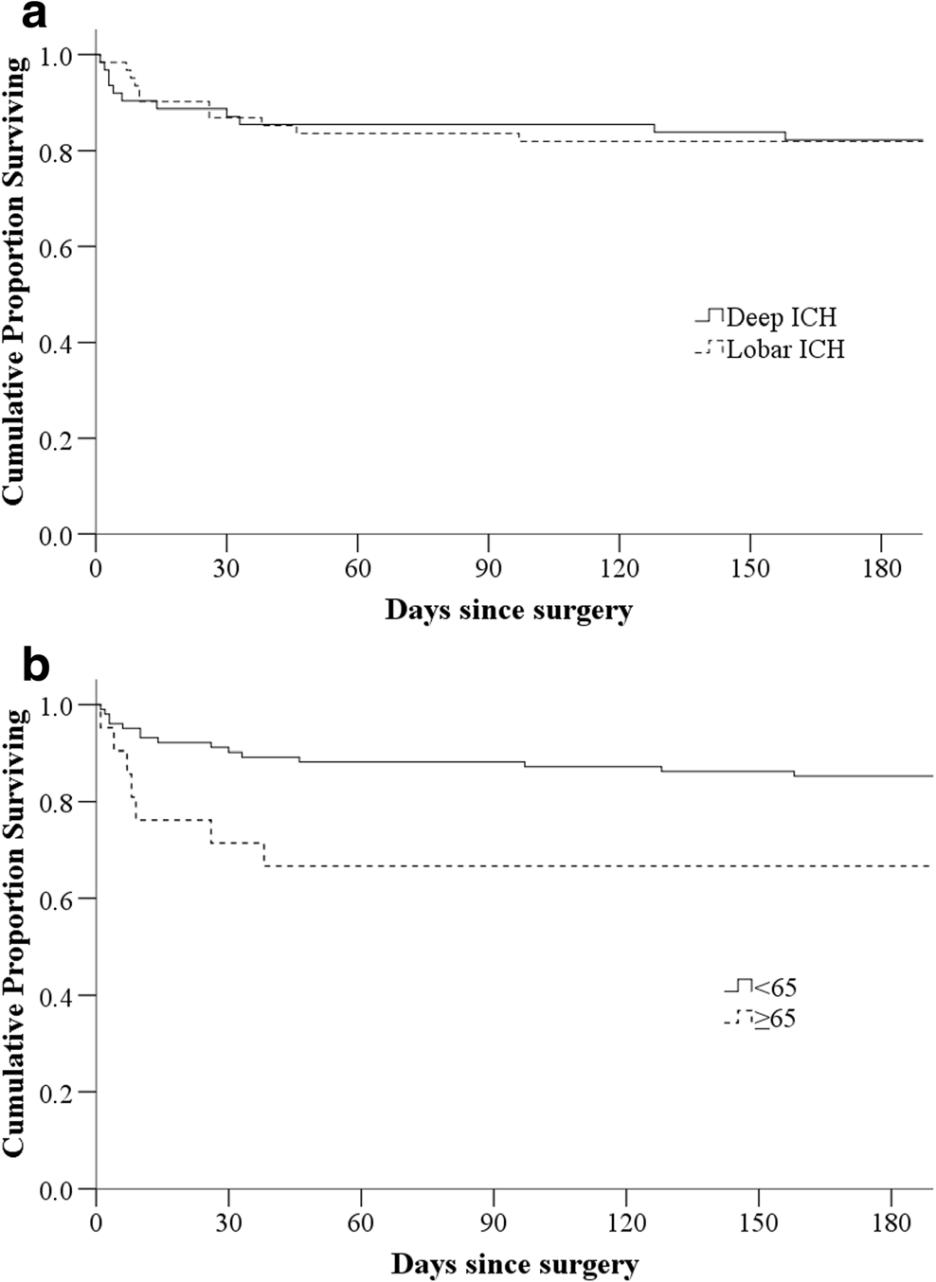
Kaplan-Meier curve showing survival at 6 months after surgery. **a** Stratified according to intracerebral hemorrhage (ICH) location. **b** Stratified by age groups (≥ 65 and < 65). Twenty-one patients ≥ 65 years old were included in the study

**Fig. 2:**
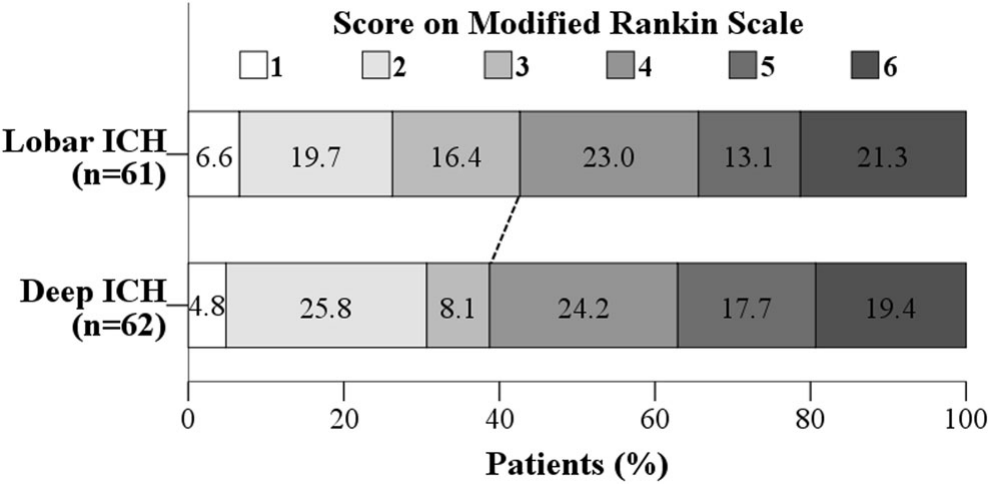
Functional outcome (modified Rankin Scale) according to intracerebral hemorrhage (ICH) location. Distribution of modified Rankin Scale (mRS) score comparing lobar and deep ICH. The dashed line separates unfavorable (mRS > 3 mRS) from favorable (≤ 3 mRS) outcome

The overall mortality at 30 days and 6 months was 13.0% and 17.9%, respectively, although higher in patients ≥ 65 years old (*p* = 0.020). Thirty-day mortality was 12.9% in patients with deep-seated ICH and 13.1% in lobar ICH (*p* = 0.972). At discharge, the median mGCS score improved to 6.0 (IQR 5–6; *p* = 0.008), and this improvement was seen both in lobar and deep-seated ICH patients. At 6 months post-surgery, 51 patients (82.3%) in the deep and 50 patients (82.0%) in lobar ICH group were alive (Fig. 1). At the final follow-up (range 8–89 months), 25 patients (20.3%) were dead with no difference between deep and lobar ICH [deep, 12/62 (19.4%); lobar, 13/61 (21.3%); *p* = 0.965] (Fig. 2).

**Fig. 3:**
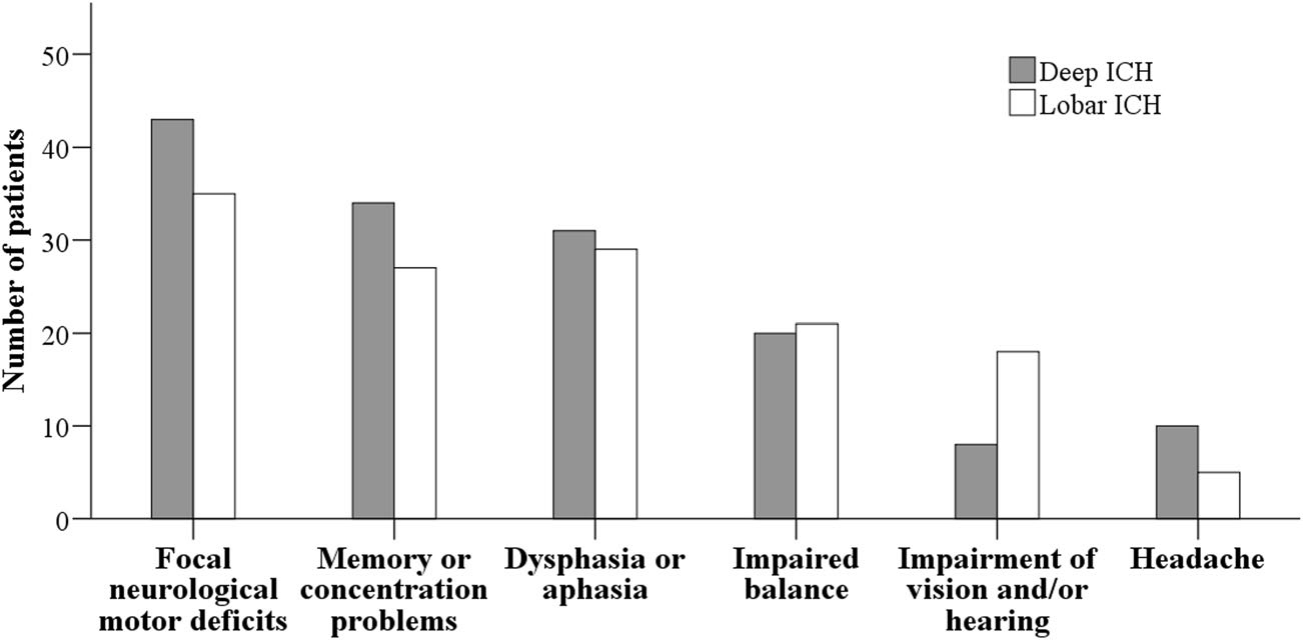
Neurological signs and symptoms at long-term follow-up

Long-term outcome was assessed in all operated ICH patients for vital status and dependence, at a mean of 4.2 ± 0.2 years after admission. Median mRS was 4.0 (IQR 2–5) for all patients. Of all included patients, 50 (40.7%) had a favorable outcome at follow-up, while 73 (59.3%) had a poor outcome. Among the survivors, 51.0% had a favorable outcome at follow-up and this proportion did not change by ICH location (mRS 0–3, 48.0% in deep ICH vs. 54.1% in lobar ICH; *p* = 0.552) (Fig. 2). Forty-three survivors (86.0%) with deep-seated ICH and 35 (72.9%) with lobar ICH had focal neurological deficits such as weakness or paralysis in an arm and/or a leg at long-term follow-up, without this difference being statistically significant (*p* = 0.136). No differences between the groups regarding long-term memory or concentration problems (*p* = 0.298), dysphasia or aphasia (*p* = 0.872), balance-related symptoms (*p* = 0.707), and severe headache (*p* = 0.263) were found. However, the proportion of patients with long-term vision and/or hearing problem impairments was significantly higher in the lobar than in the deep-seated ICH group (18 [37.5%] vs. 8 [16.0%]; *p* = 0.022) (Fig. 3).

In the age-matched analysis, 44 lobar and 44 deep ICH patients were included, with no difference in average age (lobar, 54.9 ± 9.0 years; deep, 54.6 ± 8.4 years; *p* = 0.884). Early mortality did not differ between the two groups (30 days, *p* = 0.739; 6 months, *p* = 0.772). In addition, no statistical difference was noted regarding long-term functional outcome (*p* = 0.191) between the two groups (Table 4).

**Table 4:** Postoperative outcome using 44 age-matched patients from the lobar and deep-seated ICH groups

Postoperative outcomes	Deep ICH (*n* = 44; age, 54.6 ± 8.4)	Lobar ICH (*n* = 44; age, 54.9 ± 9.0)	*p* value
30-day mortality	6 (13.6%)	4 (9.1%)	0.739
6-month mortality	8 (18.2%)	6 (13.6%)	0.772
Long-term functional outcome (≤ 3 mRS)	14 (31.8%)	21 (47.7%)	0.191

Data are presented as number (%) of patients; age in years as mean ± SD

*ICH* intracerebral hemorrhage, *mGCS* motor component of Glasgow Coma Scale score

### Predictive factors

In multivariable logistic regression, independent predictors for poor long-term outcome in the lobar ICH group were age, hemorrhage volume, preoperative mGCS score, and pupillary reactions (miosis or mydriasis, unequal pupil size, and pathological pupillary light response). In univariate logistic regression, only age was associated to outcome in the deep-seated ICH group (Tables 5 and 6).

**Table 5:** Clinical data in relation to functional outcome (modified Rankin Scale) at follow-up for the intracerebral hemorrhage (ICH) patient groups

Characteristics	Deep ICH		*p* value	Lobar ICH		*p* value
	mRS score (at follow-up)			mRS score (at follow-up)		
	0–3	4–6		0–3	4–6	
Age (years)	46.4 ± 9.9	56.4 ± 5.6	*< 0.001*	55.3 ± 10.0	60.8 ± 8.9	0.027
mGCS immediately preoperatively	5 (5–5)	5 (4–6)	0.642	6 (5–6)	5 (5–5)	*< 0.001*
Pupillary reactions (*n*)						
Normal	10 (50.0%)	10 (50.0%)	0.208	20 (64.5%)	11 (35.5%)	*< 0.001*
Pathological	14 (33.3%)	28 (66.7%)		6 (20.0%)	24 (80.0%)	
Hemorrhage volume (ml)	74.2 ± 42.3	85.8 ± 37.9	0.266	69.6 ± 21.7	102.2 ± 33.3	*< 0.001*
Time from ictus to surgery (h)						
< 24 h	19 (38.0%)	31 (62.0%)	0.815	9 (25.0%)	27 (75.0%)	*< 0.001*
≥ 24 h	5 (41.7%)	7 (58.3%)		17 (68.0%)	8 (32.0%)	

Data are presented as number (%) of patients, mean ± SD, or median (IQR). The statistically significant variables (*p* < 0.05) are shown here. Gender, presence of hypertension, diabetes or alcoholism, mean arterial blood pressure and blood glucose levels on admission, body mass index, hemisphere, length of stay, intraventricular extension, number received EVD, period with EVD, and days of EVD drainage were not associated with clinical outcome

*h* hours, *mGCS* motor component of Glasgow Coma Scale score. Pupillary pathology denotes unequal pupil size, pathological pupillary light response, miosis, or mydriasis

**Table 6:** Logistic regression analysis of clinical data in relation to long-term functional outcome (dichotomized modified Rankin Scale as dependent variable)

Characteristics	Simple logistic regression		Multiple logistic regression	
	OD (95% CI)	*p* value	OD (95% CI)	*p* value
Factors associated with favorable vs. bad outcome at follow-up in lobar ICH group (*n* = 61)				
Patient age (years)	1.067 (1.004–1.134)	*0.036*	1.108 (1.006–1.221)	*0.037*
Pupillary reactions (normal vs. pathological)	8.250 (2.336–29.139)	*< 0.001*	5.576 (1.049–29.630)	*0.044*
Preoperative mGCS (per point)	0.379 (0.174–0.827)	*0.015*	0.464 (0.220–0.977)	*0.043*
Hemorrhage volume (ml)	1.048 (1.020–1.077)	*< 0.001*	1.045 (1.006–1.085)	*0.022*
Time from ictus to surgery (< 24 h vs. ≥ 24 h)	0.157 (0.051–0.485)	*< 0.001*	0. 300 (0. 062–1.449)	0.134
Factors associated with favorable vs. bad outcome at follow-up in deep ICH group (*n* = 62)				
Patient age (years)	1.186 (1.082–1.300)	*< 0.001*	–	–

*h* hours, *mGCS* motor component of Glasgow Coma Scale score, *ICH* intracerebral hemorrhage, *OD* odds ratio. A *p*-value <0.05 is indicated by italics

## Discussion

This single-center retrospective study aimed to compare baseline characteristics, early mortality, long-term functional outcome, and prognostic factors in patients with lobar vs. deep-seated ICH who underwent surgical evacuation. Although patients with deep-seated ICH were in worse neurological status preoperatively and required more frequently EVD as well as longer ventilatory support, neither early mortality nor long-term functional outcome differed between the two groups. In lobar ICH, independent predictors of long-term outcome were age, hematoma volume, preoperative level of consciousness, and pupillary reaction, whereas only age-predicted outcome in deep-seated ICHs.

While ICHs located in deep-seated regions are more frequent than in other locations [25, 38, 45], an equal number of deep-seated as lobar ICHs were operated in our present study. Since deep-seated ICH patients were younger and more frequently in a poor neurological state, surgery was performed as a life-saving measure. Lobar ICH patients presented at our neurosurgical department were on average older, had less pupillary abnormalities, and were more likely to be in a better neurological state when compared to those with deep-seated ICHs. Older patients and in particular those with low volume basal ganglia hemorrhages without intraventricular extension were not considered candidates for surgery [17, 41], indicating that our present series is a representative cohort of surgically treated ICH patients.

The first STICH trial included 1033 patients that were randomized into two groups: early (within 24 h after randomization) surgical intervention or initial conservative treatment. A subgroup analysis suggested some benefit of surgery for superficial lobar although not deep-seated ICHs [27]. These findings led to the STICH II trial, designed to investigate whether early surgery would improve outcomes in superficial lobar ICH. This trial reported a low, 18%, mortality rate at 6 months for superficial lobar hemorrhages without IVH, in contrast to the first STICH trial which showed an overall mortality rate of 36% at 6 months for both lobar and deep ICH groups [28]. A meta-analysis evaluating previous randomized controlled trials suggested that early surgery, within 24 h from onset of the hemorrhages, can be beneficial [14].

In the present study, only 33% of all ICH patients underwent surgery later than 24 h post-ictus. Most patients (75%) with lobar ICHs operated earlier than 24 h post-ictus had a poor long-term outcome, in contrast to 32% operated later than 24 h post-ictus. This is contrary to recently published data showing that time from ictus to surgery did not significantly influence long-term outcome. In our practice, the indication for lobar ICH evacuation was based on a combination of reduced level of consciousness and/or large ICH volume, factors also negative prognostic predictors. The common presence of both a large ICH volume and an impaired level of consciousness in these patients may be a plausible explanation for the worse outcome in those operated early. In addition, deep-seated ICHs were evacuated on average 6 h earlier than lobar ICHs. In deep-seated ICHs, outcome was not influenced by timing for surgery. Emergent surgery was always conducted in patients deteriorating and/or with decreased level of consciousness, regardless of ICH location, suggesting that major improvement in outcome accomplished by even earlier surgery in our cohort is unlikely.

A large ICH volume has repeatedly been considered a negative prognostic factor and a powerful predictor of mortality [7, 40]. In addition, intraventricular extension and patient age have also, to a varying extent, been considered important prognostic factors [1, 21–23, 40, 47]. In the present study, the mean ICH volume was > 80 ml, similar between the two locations. Intraventricular extension of the ICH was more common in patients with deep-seated ICH, who also more frequently received an EVD.

ICH appears less fatal in younger individuals particularly in those surgically treated [21, 22]. In a recent study, surgical evacuation was associated with lower 3-month mortality (9.9% vs. 23.0%) in younger (16 to 49 years old) compared to ICH patients older than 49 years of age [22]. In our present study, age was associated with long-term outcome in both lobar and deep-seated ICH with a similar outcome between the two groups. Overall mortality at 30 days and 6 months post-ictus was 13.0% and 17.9% respectively, although higher in patients ≥ 65 years old in both ICH groups. At 6 months, 101 patients (82.1%) were alive which did not change significantly at the last follow-up (79.7%). Since lobar ICH patients were on average older and age was found to influence outcome in both ICH groups, we conducted an age-matched analysis that showed no significant difference in early mortality and long-term outcome between the two groups. These findings are consistent with another study showing no association between ICH location and early mortality in young surgically treated ICH patients [21]. Although mortality and morbidity rates are likely influenced by the general status of the patient including increased medical complications with advanced age, the low mortality beyond 6 months post-ictus in the present cohort argues for a stable medical condition in long-term ICH survivors.

One potential factor responsible for the failure of surgical trials is the additional brain injury inflicted by the surgery [6, 48]. Most previous trials have not been able to observe a reduced mortality in surgically treated ICH patients when compared to those receiving medical therapy [5, 20, 27, 28, 49]. In a previous study, ICH location was found to influence mortality and quality of survival when comparisons were made between subcortical and putaminal hemorrhages [30]. In the present study, no significant correlation was noted between ICH location and functional outcome. In addition, the 6-month mortality rate in lobar ICH patients was similar (18.0%) when compared to the deep-seated ICH patients (17.7%). In addition, in a subgroup analysis of 57 patients with putaminal hemorrhages, patients who underwent craniotomy and hemorrhage evacuation had better functional outcome than those conservatively treated [30]. These data, in combination with the present results, argue against the notion that surgical approach is a significant factor influencing outcome.

?twb=.27w?>As surgical and medical treatment choices for ICH continue to evolve, improved ICH outcome could result from stepwise refinement of hospital care. Admission to neurocritical care (NCC), rather than a general intensive care unit, was associated with reduced mortality in ICH patients [8]. In the present series, patients were treated by an NCC protocol previously used, e.g., in traumatic brain injury patients [9] that included the detection and monitoring of avoidable factors such as fever, high ICP, low cerebral perfusion pressure, and high glucose levels [9]. This protocol was found clinically beneficial also for patients with severe thalamic and subarachnoid hemorrhage [36, 43], and have likely contributed to the rather low mortality rate and similar outcome reported in our current study.

Patients with deep-seated ICH required more frequently EVD placement than those with lobar ICH, plausibly due to their proximity to the ventricular system, higher intraventricular hemorrhage score, and higher likelihood of hydrocephalus. The indication to proceed to EVD placement was predominantly based on radiological (ICH, hydrocephalus) features and impaired level of consciousness attributed to the intraventricular hemorrhage. In our department, an EVD is also liberally inserted in patients with a decreased level of consciousness, irrespective of the radiological findings. In the present study, long-term outcome was not associated with the duration of EVD or days of CSF drainage in either ICH group. Furthermore, outcome was similar in the two groups regardless of EVD placement and the need for CSF drainage.

The retrospective design is an obvious limitation of the present study. In addition, the STICH II trial was published in 2013, and although no marked change to the surgical selection criteria followed, we cannot exclude that the STICH II results influenced the criteria towards a more aggressive surgical approach to lobar ICH patients. The clinical decisions may also differ slightly among the treating neurosurgeons. Thus, some selection bias may exist. However, in our department, there is a general agreement on the factors influencing surgical decisions as well as when to refrain from surgery such as in very large ICHs, deeply comatose patients with or without marked pupil abnormalities, in the elderly, and in patients with severe co-morbidities. Unfortunately, we were unable to assess the prognostic role of the remaining and/or recurrent ICH post-operatively. However, since the number of re-operations was low, a marked influence of the remaining ICH volume is unlikely. Moreover, subtle differences such as quality of life end points were not displayed in the chosen dichotomized mRS outcome measure, since the present study could only assess mortality and long-term functional outcome. Since a proportion of survivors had an unfavorable outcome, quality of life measures are important and need to be addressed in future studies. However, the present results were obtained in a busy neurosurgical center with a large patient catchment area where, to the extent possible, strict selection criteria for surgery were applied. Surgical technique was also uniform, adding to the applicability of our results. There are numerous ethical arguments against a strict RCT in the surgical management of ICH patients since many surgeries are performed as life-saving measures. Thus, large descriptive outcome studies are needed to better define the value and indications for surgery in the treatment of ICH.

## Conclusion

Despite large hemorrhage volumes in both ICH locations and the heterogeneous clinical characteristics of the patient cohort, neither mortality nor long-term functional outcome differed between the ICH locations. In both groups, approximately half of the surviving patients had a good functional outcome at long-term follow-up regardless of their preoperative state. Our data imply that the hesitance to proceed to surgery in deep-seated ICHs may not be warranted, particularly in younger patients with deteriorating level of consciousness.
